# Fluid chemistry alters faunal trophodynamics but not composition on the deep-sea Capelinhos hydrothermal edifice (Lucky Strike vent field, Mid-Atlantic Ridge)

**DOI:** 10.1038/s41598-024-52186-1

**Published:** 2024-01-22

**Authors:** Joan M. Alfaro-Lucas, Daniel Martin, Loïc N. Michel, Agathe Laes, Cécile Cathalot, Sandra Fuchs, Jozée Sarrazin

**Affiliations:** 1 Univ Brest, Ifremer, CNRS, Unité BEEP, 29280 Plouzané, France; 2 Centre d’Estudis Avançats de Blanes (CEAB-CSIC), Blanes, Catalonia Spain; 3 Université de Liège, Liège, Belgium; 4 Present Address: Department of Biology, University of Victoria, Victoria, BC Canada

**Keywords:** Biodiversity, Community ecology

## Abstract

The recently discovered deep-sea Capelinhos hydrothermal edifice, ~ 1.5 km of the main Lucky Strike (LS) vent field (northern Mid-Atlantic Ridge), contrasts with the other LS edifices in having poorly-altered end-member hydrothermal fluids with low pH and chlorine, and high metal concentrations. Capelinhos unique chemistry and location offer the opportunity to test the effects of local abiotic filters on faunal community structure while avoiding the often-correlated influence of dispersal limitation and depth. In this paper, we characterize for the first time the distribution patterns of the Capelinhos faunal communities, and analyze the benthic invertebrates (> 250 µm) inhabiting diffusive-flow areas and their trophic structures (δ^13^C, δ^15^N and δ^34^S). We hypothesized that faunal communities would differ from those of the nearest LS vent edifices, showing an impoverished species subset due to the potential toxicity of the chemical environment. Conversely, our results show that: (1) community distribution resembles that of other LS edifices, with assemblages visually dominated by shrimps (close to high-temperature focused-fluid areas) and mussels (at low-temperature diffuse flow areas); (2) most species from diffuse flow areas are well-known LS inhabitants, including the bed-forming and chemosymbiotic mussel *Bathymodiolus azoricus* and (3) communities are as diverse as those of the most diverse LS edifices. On the contrary, stable isotopes suggest different trophodynamics at Capelinhos. The high δ^15^N and, especially, δ^13^C and δ^34^S values suggest an important role of methane oxidation (i.e., methanotrophy), rather than the sulfide oxidation (i.e., thiotrophy) that predominates at most LS edifices. Our results indicate that Capelinhos shows unique environmental conditions, trophic structure and trophodynamics, yet similar fauna, compared to other LS edifices, which suggest a great environmental and trophic plasticity of the vent faunal communities at the LS.

## Introduction

Understanding the processes structuring faunal assemblages is at the core of community ecology^1^. Regional species pools are determined by speciation, extinction and dispersion interacting with smaller-scale abiotic and biotic processes that ultimately determine species coexistence and community structure at local habitats^2–4^. Assessing these community assembly processes is key to predict disturbance impacts on, and resilience of, ecosystems^5^.

In 1977, lush taxon-novel communities fueled by in situ microbial chemoautotrophy were discovered in the Galapagos Rift hydrothermal vents at 2550 m depth^6,7^. Deep-sea vents are often dominated by large foundation chemosymbiotic species not found elsewhere, which promote the establishment of smaller invertebrates^8,9^. Overall, this gives rise to highly productive, biomass-rich communities, orders of magnitude denser than those of the surrounding, energy-limited deep sea (reviewed in^10–12^). More than four decades of exploration have revealed hundreds of active vent fields patchily distributed along diverse tectonic settings in the world oceans^13^. However, all together these numerous island-like habitats occupy only 50 km^2^ worldwide^14^. Vents are thus small natural features with a biogeochemical and ecological relevance disproportionate to their size^14^. On top of that, our knowledge of the complex processes driving community assembly in vent ecosystems is still insufficient^11^. This knowledge gap is worrisome given the current context where climate change, pollution and deep-sea mining could potentially trigger adverse impacts on vents habitats in the near future^15–17^.

Vent communities differ across tectonic settings, constituting different biogeographic provinces covering entire, or parts of, ocean basins^10,18–20^. Regionally, larval dispersal connects the network of island-like vent habitats separated by 10–100 s of kilometers, but topographic barriers and oceanic circulation regimes influence connectivity^21–26^. At a more local scale, vent fields rarely harbor all province species, and significant faunal dissimilarities have been observed even between neighboring sites^27–30^. Differences may be driven by local environmental filters, such as distinct fluid chemistry and substratum nature^28,29,31,32^ and/or by intricate biological processes^33–36^. More complex patterns occur in arc and back-arc basin vents (e.g.,^30,37^) and at slower-spreading mid-oceanic ridges (e.g.,^27,38^) where geology is more heterogeneous.

**Figure 1:**
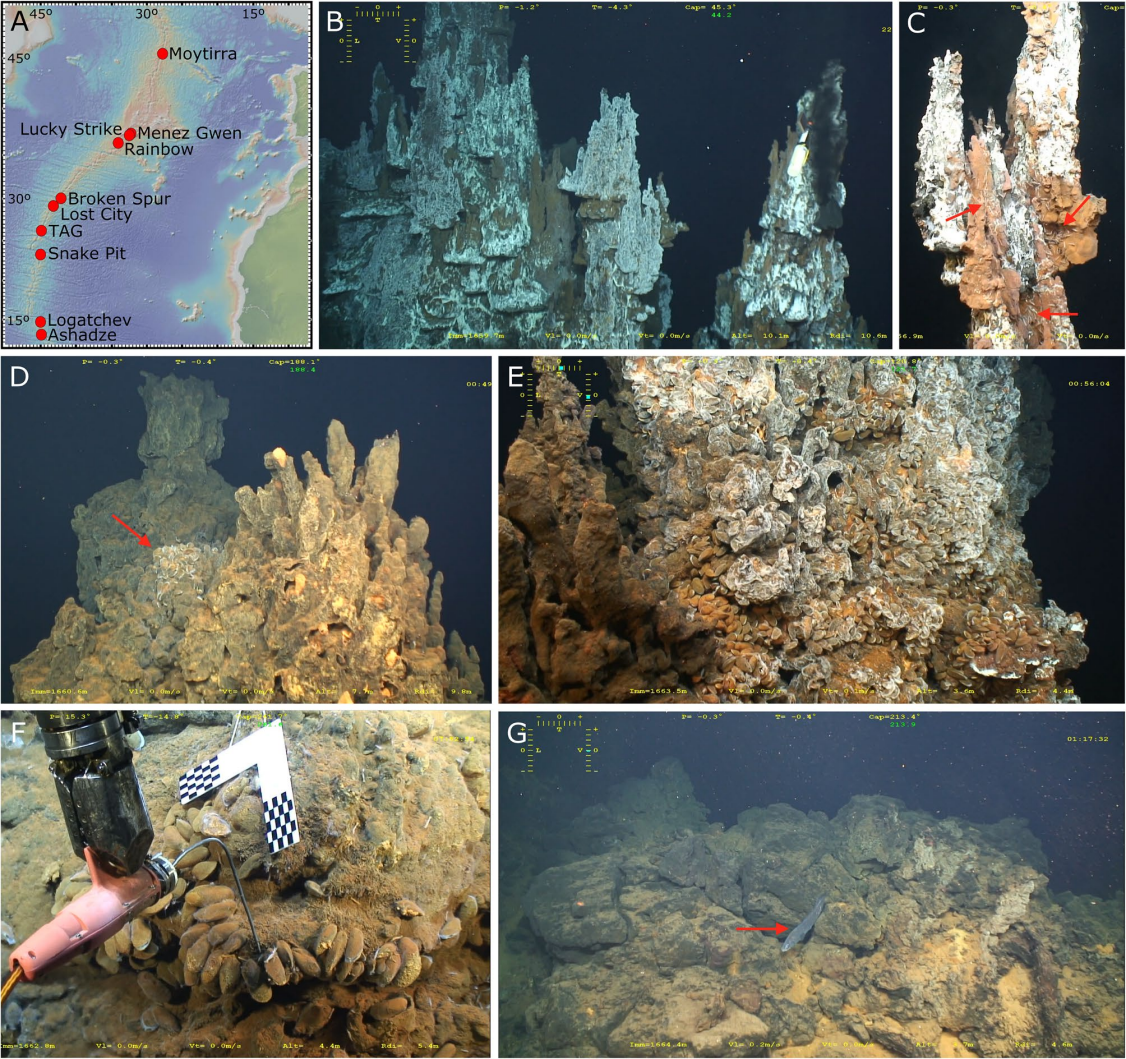
**(A)**. Main hydrothermal fields in the northern Mid-Atlantic Ridge; Capelinhos is located ~ 1.5 km off the main Lucky Strike field. Capelinhos habitats. (**B)**. Active and inactive black smokers with microbial mats at the summit of the structure. (**C)**. Shrimp aggregations (red arrows) probably belonging to *Mirocaris fortunata* on top of a black smoker. (**D)**. Top of a senescent chimney with a patch of *Bathymodiolus azoricus* mussels (red arrow) in between its two heads. (**E)**. Dense *B. azoricus* beds and microbial mats at the base of a senescent chimney. F. Patch of large *B. azoricus* with sparse shrimps; the CHEMINI analyzer inlet and our checkerboard calibrated target are visible. (**G)**. *Cataetyx*-like fish (red arrow) on sulphide rubble surroundings. Map made with GeoMapApp 3.6.15 (www.geomapapp.org)/CC BY/CC BY (Ryan et al.^51^).

In the slow-spreading northern Mid-Atlantic Ridge (nMAR) (Fig. 1A), visually-dominant species assemblages differ between vent fields, creating a complex community mosaic along the ridge^27,28,39–41^. Generally, shrimps dominate higher-temperature habitats. *Mirocaris fortunata* (Martin & Christiansen, 1995) dominates the Moytirra, Menez Gwen and Lucky Strike vents, and *Rimicaris exoculata* Williams & Rona, 1986 dominates deeper vents such as TAG, Snake Pit or Rainbow^28,40,42^. Intermediate temperature habitats appear to be dominated by newly described gastropod assemblages including two different species: *Lepetodrilus atlanticus* Warén and Bouchet 2001 at Menez Gwen and *Peltospira smaragdina* Warén and Bouchet 2001 at seven other deeper vent fields^43^. At lower-temperature habitats, chemosymbiotic mussels are the main foundation species. The shallower Menez Gwen and Lucky Strike vent fields (850–1700 m depth) are dominated by *Bathymodiolus azoricus* Cosel & Comtet, 1999, which is less abundant at the deeper and ultramafic-hosted Rainbow field (2300 m depth)^28,44^. Broken Spur (3100 m depth) is a hybridizing zone between *B. azoricus* and *Bathymodiolus puteoserpentis* Cosel, Métivier & Hashimoto, 1994 the latter exclusively dominating Snake Pit (3500 m) and Logatchev (3000 m depth) fields^27,40,45^. At TAG (3670 m depth), the metal-enriched fluids are hypothesized to prevent mussel establishment^27,46^. Mussels are also absent at Moytirra (2900 m depth), dominated by the gastropod *P. smaragdina* Warén & Bouchet 2001, and Ashadze-1 (4200 m depth)^42,47^. Adding more complexity to this mosaic, Lost City ultramafic-hosted vents (800 m, ~ 15 km off axis) do not support the typical dense vent shrimp/mussel assemblages^48^, although evidence suggest it did so in the past^49,50^. Given this multifaceted picture and despite the 46 years of vent research, the processes driving the community assembly at nMAR vents are just starting to be resolved.

Here, we characterize for the first time the distribution, composition and trophic structure of the Capelinhos vent communities, particularly those associated to mussel beds, and compare them to those of other Lucky Strike (LS) edifices and to several nMAR vents. Capelinhos is a recently-discovered, isolated hydrothermal structure located off the main LS field (nMAR) (Fig. 1A)^52^. Capelinhos emits poorly-altered end-member fluids with low pH and chlorine, and high metal concentrations. In contrast, the other LS edifices show intermediate (e.g., Eiffel Tower, Montségur) or highly-altered fluids (e.g., Crystal) with higher pH and lower metal concentrations^52,53^. The proximity and similar depth of Capelinhos to other LS edifices create a natural experiment restricting the effects of dispersal limitation and depth-related processes while isolating the putative influence of local abiotic conditions on faunal communities. We hypothesize that the potentially harsher chemistry, i.e., low pH and high metal concentrations, of Capelinhos could act as environmental filter, influencing the fauna distribution and composition, and preventing the establishment of some species, compared to other LS edifices.

## Results

### Habitat characterization based on ROV imagery

Capelinhos edifice has a steep profile with two main structures (Fig. 1, Supplementary material Video S1). The first structure has several “candelabra-like” chimneys, some emitting highly-focused hydrothermal fluids (Fig. 1B,C, Supplementary material Video S1–1:30 min). These chimneys are densely colonized by white microbial mats and sparse shrimp aggregations (probably *M. fortunata*) close to the focused fluids (Fig. 1C, Supplementary material Video S1–5:00 min). Dense mussel aggregations (including patches of juveniles) and microbial mats occur at the base. The second structure is a single, large, senescent Y-shape chimney. The limited diffuse-flow area found at the top is colonized by a small patch of large mussels (Fig. 1D, Supplementary material Video S1–7:13 min). The base of this structure is colonized by dense mussel beds (covered or not by microbial mats), especially on one of its sides (Fig. 1E, Supplementary material Video S1–8:40 min). The few flanges at the base of each main structure are colonized by large mussels and shrimps (Fig. 1F, Supplementary material Video S1). Crabs, probably *Segonzacia mesatlantica* (Williams, 1988), and shrimps were present in between and over the mussels (Fig. 1F, Supplementary material Video S1–9:36). Some fishes occur at the vicinity of the chimneys and in peripheral areas (Fig. 1G, Supplementary material Video S1–10:00 min).

### Environment, community composition and species diversity

Temperatures over mussel assemblages range from 4.32 to 9.88 °C (Table 1 and Supplementary material Fig. S1). Between-sample variability is relatively high, despite the close proximity of the probes (~ 50 cm), as observed for Fe(II) (0.98–2.40 µM) and even more for ƩS (21.80–44.8 µM) (Table 1; Supplementary material Table S2 and Fig. S1). Compared with the habitats colonized by *B. azoricus* at the Eiffel Tower edifice, the temperatures at Capelinhos are similar to those in cold and intermediate-temperature habitats, while the high Fe(II) and, especially, ƩS concentrations are more similar to those of the warm habitats, highlighting Capelinhos distinct fluid chemistry (Table 1, Supplementary material Table S2 and Fig. S2).

**Table 1:** Temperature (T, °C), iron (Fe(II), µM) and sulfide (ƩS, µM) concentrations (mean ± standard deviation) in *Bathymodiolus azoricus* mussel bed samples at Capelinhos.

		T			Fe(II)	ƩS
		Mean	Min	Max		
Capelinhos		5.68 ± 0.67	4.32	9.88	1.77 ± 0.58	31.88 ± 9.47
Eiffel Tower	Cold	5.11 ± 0.37	NA	NA	0.54 ± 0.37	3.31 ± 1.94
	Intermediate	6.04 ± 0.59	NA	NA	1.13 ± 0.98	12.83 ± 6.28
	Warm	7.59 ± 1.97	NA	NA	3.33 ± 2.36	32.38 ± 19.16
Montségur		5.2	4.6	6.1	0.2 ± 0.1	2.7 ± 0.2
		6.9	5.1	11.5	1.1 ± 0.3	3.1 ± 1
		9.5	6.1	22.1	2.2 ± 0.2	2.3 ± 0.2
		5.5	5.1	11.4	0.2 ± 0.1	3.2 ± 2.7
		5.3	4.6	7.1	0.6 ± 1.1	0.9 ± 0.2

Environmental information of *B. azoricus* cold, intermediate and warm microhabitats of the Eiffel Tower^44^ edifice and samples from Montségur edifice^54^ (both located at Lucky Strike) are provided for comparison. Mean temperature associated to Fe(II) and ƩS measurements at Capelinhos were 4.75 ± 0.57 and 5.04 ± 0.30 °C, respectively (see raw data in Supplementary material Table S2 and Fig. S2).

**Figure 2:**
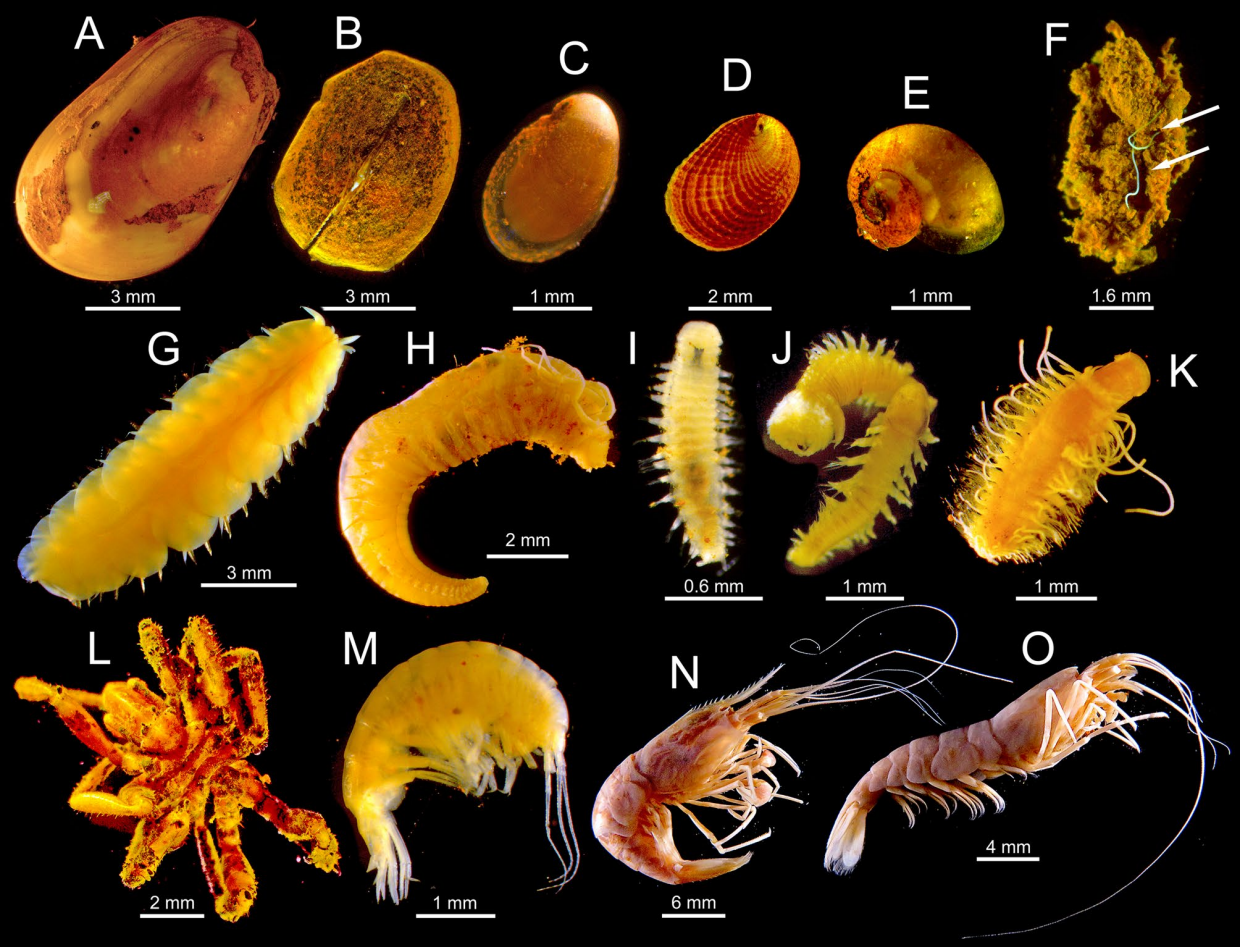
Main macrobenthic invertebrate species inhabiting Capelinhos. (**A**) *Bathymodiolus azoricus.* (**B**) *Pseudorimula midatlantica*. (**C**) *Lepetodrilus atlanticus*. (**D**) *Divia briandi*. (**E**) *Protolira valvatoides*. (**F**) *Oncholaimus dyvae* (arrow) within *A*. *lutzi* tube. (**G**) *Branchipolynoe seepensis*. (**H**) *Amphisamytha lutzi*. (**I**) *Ophryotrocha fabriae*. (**J**) *Glycera tesselata*. (**K**) Hesionidae sp. (**L**) *Sericosura* sp. (**M**) *Bouvierella curtirama*. (**N**) *Alvinocaris markensis*. (**O**) *Mirocaris fortunata.*

We have identified 1986 individuals from 28 species/morphospecies representing five phyla (Table 2, Fig. 2, Supplementary material Table S3). Genetic analyses confirm the dominant species as belonging to *B. azoricus* (https://sextant.ifremer.fr/Donnees/Catalogue#/metadata/72f13a1b-3770-4108-828c-b12aa4249987). Following dominant species are the tubicolous annelid *Amphisamytha lutzi* (Desbruyères & Laubier, 1996) and dirivultid copepods (Table 1; Fig. 2). The annelid *Branchipolynoe seepensis* Pettibone, 1986 and the nematode *Oncholaimus dyvae* Zeppilli et al., 2019 were also abundant, often occurring inside *B. azoricus* and within *A*. *lutzi* matrix tubes, respectively (Fig. 2B,G).

**Table 2:** Relative mean (± sd) abundance (N) and isotopic composition (δ^13^C, δ^15^N and δ^34^S) of species found at the Capelinhos hydrothermal structure, 1.5 km off the Lucky Strike vent field (nMAR).

Phylum	Class	Order	Species	N	δ^13^C	δ^15^N	δ^34^S	ET
Annelida	Polychaeta	Terebellida	*Amphisamytha lutzi*	18.76 ± 2.28	− 17.16 ± 1.60 (15)	3.07 ± 1.57 (15)	11.26 ± 1.05 (15)	S
		Phyllodocida	*Glycera tesselata*	0.03 ± 0.05	− 21.84	9.11	–	S
			*Branchipolynoe seepensis*	8.91 ± 4.76	− 20.75 ± 3.04 (6)	− 3.10 ± 3.71 (6)	8.71 ± 0.42 (6)	S
			*Branchipolynoe* sp. 2	0.12 ± 0.20	− 19.18	− 1.15	9.72	–
			Polynoidae sp. 3	0.48 ± 0.50	–	–	–	–
			*Branchinotogluma* sp.	0.47 ± 0.15	− 23.61 ± 6.89 (3)	6.68 ± 1.06 (3)	10.23 ± 0.98 (3)	–
			*Branchinotogluma* sp. 2	0.03 ± 0.05	–	–	–	–
			Hesionidae sp.	0.20 ± 0.35	–	–	–	–
		Eunicia	*Ophryotrocha fabriae*	2.56 ± 0.96	− 24.88	2.55	–	S
Arthropoda	Hexanauplia	Harpacticoida	*Smacigastes micheli*	0.30 ± 0.30	–	–	–	S
			Ameiridae sp. 1	5.78 ± 5.09	–	–	–	M
			Miraciidae sp.	3.47 ± 4.37	–	–	–	M
		Siphonostomatoida	Dirivultidae sp.	18.21 ± 3.24	− 19.37 ± 0.16 (2)	0.75 ± 0.36 (2)	9.40 ± 0.38 (2)	M
	Malacostraca	Amphipoda	*Luckia striki*	0.09 ± 0.15	− 20.18	4.46	–	S
			*Bouvierella curtirama*	0.65 ± 0.57	− 19.06 ± 0.01 (2)	2.80 ± 0.51 (2)	10.91 ± 2.30 (2)	S
			Amphipod sp. 1	0.03 ± 0.05	− 18.75	3.27	–	–
		Decapoda	*Mirocaris fortunata*	2.85 ± 3.10	− 16.72 ± 1.75 (10)	6.34 ± 0.97 (10)	10.34 ± 1.71 (10)	S
			*Alvinocaris markensis*	0.10 ± 0.17	− 23.47	2.79	8.55	S
	Ostracoda		Ostracoda sp.	2.68 ± 1.35	–	–	–	–
	Pycnogonida	Pantopoda	*Sericosura* sp*.*	0.20 ± 0.35	− 21.57	4.61	10.35	M
Mollusca	Bivalvia	Mytilida	*Bathymodiolus azoricus*	21.90 ± 3.17	− 20.95 ± 3.08 (6)	− 4.70 ± 2.53 (6)	7.97 ± 1.66 (6)	S
	Gastropoda	Lepetellida	*Lepetodrilus atlanticus*	1.50 ± 1.33	− 20.04	4.30	12.17	S
			*Pseudorimula midatlantica*	1.81 ± 1.04	− 25.99 ± 0.51 (2)	2.28 ± 0.04 (2)	8.05 ± 0.08 (2)	S
		Trochida	*Protolira valvatoides*	0.77 ± 0.67	− 22.98	3.97	11.97	S
			*Lurifax vitreus*	0.03 ± 0.05	–	–	–	S
		Cycloneritida	*Divia briandi*	0.29 ± 0.50	− 11.89	5.95	8.29	S
Nematoda	Enoplea	Enoplida	*Oncholaimus dyvae*	7.16 ± 4.40	− 18.32	6.32	9.51	S
Nemertea			Nemertea sp.	0.63 ± 0.76	− 17.23	2.04	10.85	–

ET column indicates if the species (S) or morphotype (M) is found at Eiffel Tower edifice in the main Lucky Strike (Alfaro-Lucas et al.^55^; J.M. Alfaro-Lucas *pers. obs*.). In brackets the number of samples used to estimate the isotopic composition of species.

**Figure 3:**
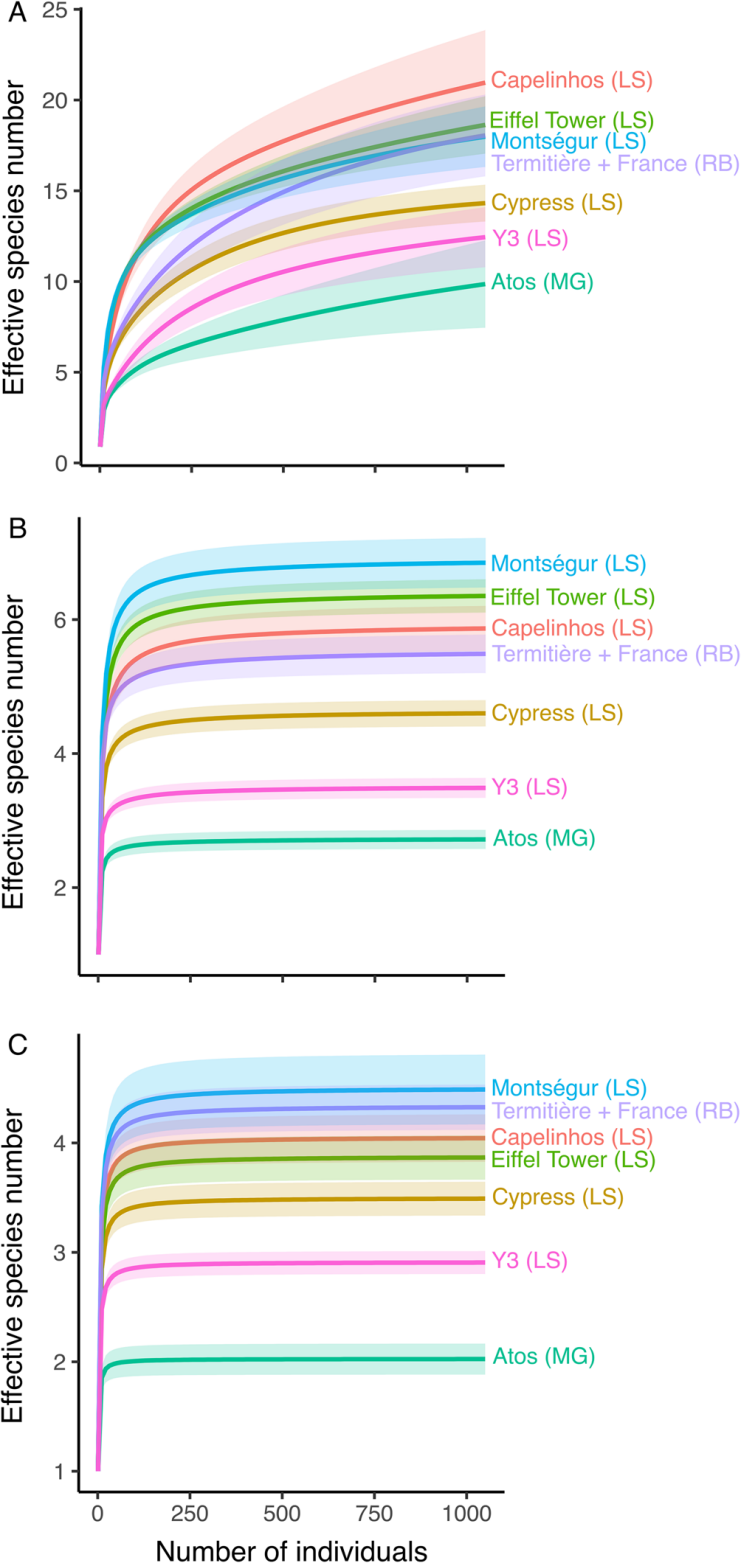
Rarefaction curves and 95% confidence intervals (shaded areas) based on the abundance of the communities at different hydrothermal edifices on the northern Mid-Atlantic Ridge. (**A**) Species richness. (**B**) Shannon index. (**C**) Simpson index. LS, Lucky Strike; MG, Menez Gwen and RB, Rainbow vent fields. LS, MG and RB data from Sarrazin et al.^44^.

**Figure 4:**
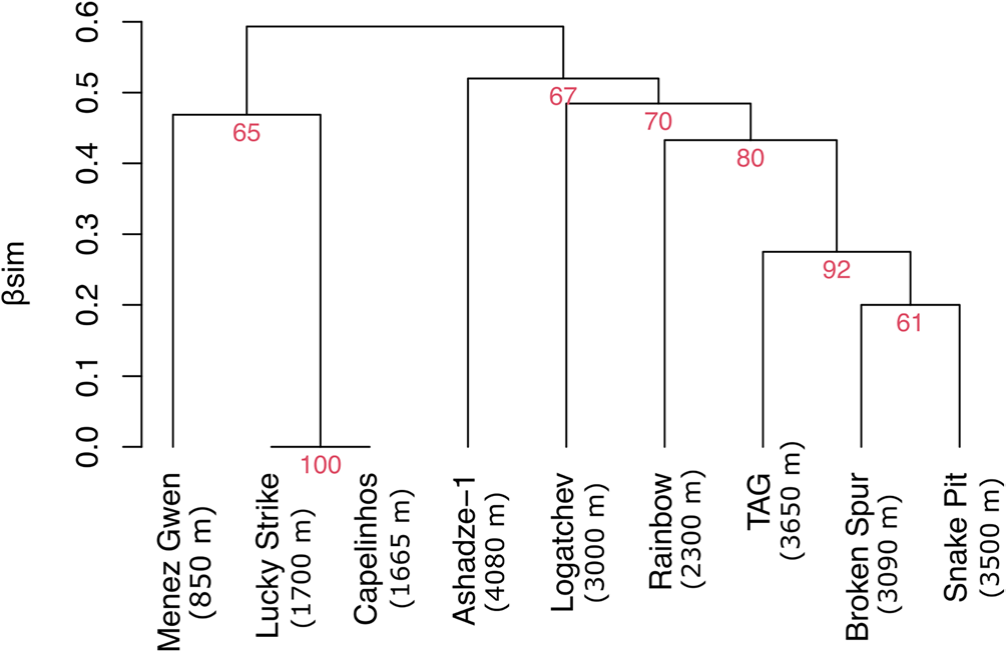
Hierarchical cluster analysis of vent communities at hydrothermal fields and Capelinhos edifice (off the main Lucky Strike vent field) on the northern Mid-Atlantic Ridge. Red numbers: Approximately Unbiased *p* value. Data modified from Boschen-Rose and Colaço^41^.

^0^D, ^1^D and ^2^D are as high at Capelinhos as at the assemblages of *B. azoricus* from Eiffel Tower and Montségur edifices (Fig. 3). β_sim_ is equal to 0 when compared to the LS vents. In other words, their assemblages do not differ in species composition from those observed at the LS (AU-*P* = 100) (Fig. 4). Capelinhos and LS cluster with the shallower Menez Gwen (850 m depth), whereas deeper vents, including Rainbow (2300 m), cluster together (Fig. 4) highlighting main faunal composition differences between MAR shallower and deeper vents.

### Stable isotopes

**Figure 5:**
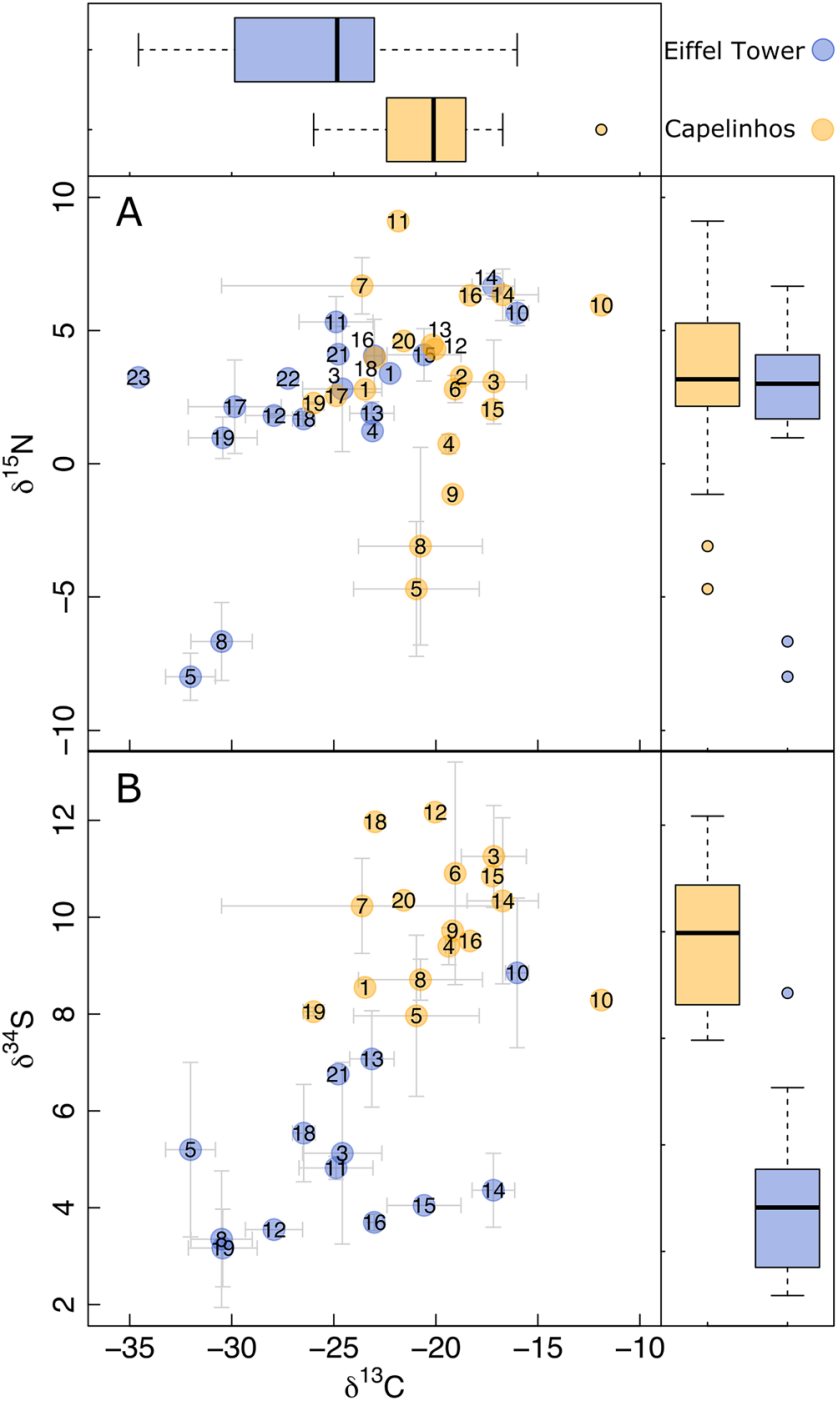
Isotopic composition of species found in Capelinhos structure and Eiffel Tower edifice (Lucky Strike vent field). (**A**) δ^13^C/δ^15^N isotopic space. (**B**) δ^13^C/δ^34^S isotopic space. Boxplots show the δ^13^C (top), δ^15^N (top right) and δ^34^S (bottom right) isotopic value distributions. 1 = *Alvinocaris markensis*, 2 = Amphipoda sp., 3 = *Amphisamytha lutzi*, 4 = Dirivultidae sp., 5 = *Bathymodiolus azoricus*, 6 = *Bouvierella curitama*, 7 = *Branchinotogluma* sp., 8 = *Branchipolynoe seepensis*, 9 = *Branchipolynoe* sp., 10 = *Divia briandi*, 11 = *Glycera tesselata*, 12 = *Lepetodrilus atlanticus*, 13 = *Luckia striki*, 14 = *Mirocaris fortunata*, 15 = Nemertea sp., 16 = *Oncholaimus dyvae*, 17 = *Ophryotrocha fabriae*, 18 = *Protolira valvatoides*, 19 = *Pseudorimula midatlantica*, 20 = *Sericosura* sp., 21 = *Lirapex costellata*, 22 = *Pseudorimula midatlantica*, 23 = *Smacigastes micheli*. Eiffel Tower data from Alfaro-Lucas et al.^56^.

Among Capelinhos fauna, mean δ^13^C is more negative in the limpet *Pseudorimula midatlantica* J. H. McLean, 1992 and less negative in the gastropod *Divia briandi* (Warén & Bouchet, 2001) (Table 1, Fig. 5A). Mean δ^15^N is lower in *B. azoricus* and higher in the polychaete *Glycera tesselata* Grube, 1863 (Table 1, Fig. 5A). Mean δ^34^S is also lower in *B. azoricus* and higher in the limpet *L. atlanticus* (Table 1, Fig. 5B). The δ^13^C/δ^15^N isotopic structure show a more-compact, rhomb-like structure (*B. azoricus* and *B. seepensis* at the bottom, the predator *G. tesselata* at the top, and the grazers *P. midatlantica* and *D. briandi* in the left and right corners, respectively) when compared to the common “upper diagonal” one of Eiffel Tower (*B. azoricus* and *B. seepensis* at the bottom and *Mirocaris fortunata* and Nemertea sp. on the top) (Fig. 5A). Mean δ^13^C and δ^34^S are significantly higher (δ^13^C: t = 3.93, DF = 36, *P* < 0.001; δ^34^S: t = 8.65, DF = 27, *P* < 0.001) at Capelinhos than at Eiffel Tower (Fig. 5A,B).

## Discussion

Contrary to our expectations, our results suggest a negligible role of the putatively more toxic chemistry of Capelinhos in the distribution and structure of faunal communities compared to those at other LS edifices. The distribution of assemblages along the hydrothermal gradient match the archetypal distribution observed in LS. For instance, shrimps, likely *M. fortunata*, where observed at the warmest habitats close to focused fluids, whereas *B. azoricus* beds were observed at lower-temperature, fluid-diffusive habitats (4.32–9.88 °C), as described for the Eiffel Tower edifice^44,57–60^. All species found at Capelinhos lower temperature diffusive-fluid habitat, including several taxa identified to genus level and/or as morphotypes (Alfaro-Lucas *pers. obs.*), inhabit the other LS edifices, sharing the dominance of *B. azoricus*^28,39,59,61^. Species abundances also suggest a similar assemblage structure at Capelinhos and at the LS Eiffel Tower and Montségur edifices^44,54,55,59^. Specifically, the dominance of *B. azoricus*, *B. seepensis* and *A. lutzi*, as observed at Capelinhos, is indicative of lower-temperature mussel-bed habitats in the Eiffel Tower edifice^59^.

The putative harsher chemical environment linked to Capelinhos fluids does not limit species diversity at mussel beds, as hypothesized, being instead comparable to those in the most diverse LS edifices^44^, as supported by the rarefied diversities. Similar results have been reported in mussel beds and tubeworm bushes on the East Pacific Rise, where the assemblages from different chemical environments exhibit similar diversities and community structures^62,63^. At LS, *B. azoricus* and its associated species colonize lower temperature-habitats within edifices with different chemistries, highlighting their environmental^44^ and trophic^64,65^ plasticity. However, iron concentrations in the end-member fluid at Capelinhos are the highest of LS (2789.4 ± 84.8 μM)^52,53^, leading to higher iron concentrations in the mixing gradient between seawater and hydrothermal fluid at Capelinhos than to other LS sites^66,67^. Very diluted environments characterized with strong lateral entrainment of seawater are trickier to characterize. Nevertheless, chemical analyses in our study revealed that Fe(II) and ƩS concentrations in cooler diffuse flow areas tend to be higher in Capelinhos than at similar areas with similar temperature and similar mussel assemblages at Eiffel Tower and Montségur edifices (LS)^54,59^. High metal concentrations are hypothesized to limit and even prevent colonization by the species of *Bathymodiolus* at some nMAR vents^27^. However, our results suggest that neither *B. azoricus*, nor the associated fauna, are limited at Capelinhos lower temperature-habitats, highlighting their ability to colonize a wide range of chemical environments. The complex 3D biogenic habitat created by *B. azoricus* likely promotes small scale turbulence and enhances fluid dilution with seawater, buffering against harsh and concentrated chemical environment and fostering a similarly structure of associated fauna^44^, as observed for other vent foundation species elsewhere^9,62,63^. The mechanisms at play are unclear, but in addition to altering environmental conditions and fluid flow, mussels could increase the habitat complexity, niche availability and productivity, among others^8,9,63,68^.

The Rainbow field (2300 m depth, ~ 200 km south from Capelinhos) also shows high concentrations of iron in end-member fluids [up to 24,050 Fe(II) (µM)]^69^, and a limited abundance of *B. azoricus*, which are covered with orange metal deposits as observed at Capelinhos (J. Sarrazin *pers. comm.*). However, species similarity was higher between Capelinhos, LS and Menez Gwen (800 m depth, ~ 100 km from LS) than between Capelinhos and Rainbow, which agrees with recently results showing higher similarity between LS and Menez Gwen^41^. This result could suggest that processes acting at broader scales, such as species dispersal limitation shaping the available colonizing species, may play a stronger role in governing the species composition of mussel assemblages along the ridge^44^ than high iron concentrations^27^. In fact, the species diversity of *B. azoricus* assemblages at Rainbow is similar to those from the richest LS edifices, including Capelinhos, reinforcing the idea that high metal concentration alone may not necessarily lead to a decrease in species diversity.

Despite the similar faunal composition and structure, stable isotopes reveal profound trophodynamic differences between Capelinhos and other LS edifices. nMAR vent communities usually present an upward-diagonal-like δ^13^C/δ^15^N structure, with two rather independent compartments: (1) *B. azoricus* and *B. seepensis* (with the most negative δ^13^C/δ^15^N isotopic values) being isolated in one end, and (2) bacterivores, detritivores, scavengers and predators (with less negative δ^13^C and positive δ^15^N isotopic values) being spread at the other extreme (e.g.,^55,64,65,70^). Conversely, Capelinhos shows: (1) a rhombus-like δ^13^C/δ^15^N structure, with *B. azoricus* and *B. seepensis* at the bottom, the predator *G. tesselata* at the top, and the grazers *P. midatlantica* and *D. briandi* in the left and right corners, respectively; (2) a not so evident isolation of *B. azoricus* and *B. seepensis* because dirivultid copepods and the *Branchipolynoe* found outside their hosts showed closed isotopic values, and (3) an overall less negative δ^13^C and more positive δ^15^N isotopic values, particularly in *B. azoricus* and *B. seepensis*. Notable exceptions are the shrimps *A. markensis* and *M. fortunata* that showed similar δ^13^C/δ^15^N isotopic composition between edifices.

Less negative δ^13^C values may be indicative of the reductive tricarboxylic acid (rTCA) cycle rather than the Calvin–Benson–Bassham (CBB) cycle^65^. However, rTCA is usually associated with vigorous-fluid flux rather than lower temperature habitats^65^. Furthermore, less negative δ^13^C and more positive δ^15^N ratios have been also observed for *B. azoricus* at Sintra, a LS edifice where methane is available in higher concentrations^71^. This suggests that methane oxidation (methanotrophy) could be an important energetic pathway at Capelinhos, in addition to CCB (thiotrophy). Although not as evident as in our study, Rainbow assemblages of *B. azoricus* show a similar rhombus-like δ^13^C/δ^15^N structure, less negative δ^13^C and more positive δ^15^N isotopic values, which have been also attributed to the higher contribution of methanotrophy^65,69^.

The δ^34^S values of Capelinhos fauna, more positive than that of Eiffel Tower, also support the methanotrophy hypothesis. Values over 10‰ are usually associated to consumption of photosynthetic organic matter^72^. However, this source has been consistently discarded in nMAR vent food webs^70^, including those at LS^55,64,65^. Furthermore, Capelinhos is roughly at the same depth and only ~ 1.5 km apart from the other LS edifices. This and the overall low δ^15^N (− 4.70 to 9.11‰) allows discarding a depth/location effect on a putative increase in photosynthetic organic matter suggesting instead chemosynthetic pathways. Methanotrophy instead of thiotrophy may lead to more positive δ^34^S values^73^ and Capelinhos fluids are naturally enriched in ^34^S, reflecting contrasting subseafloor fluid/rock interactions compared to the other LS edifices^74^. Thus, we suggest the more positive δ^34^S values at Capelinhos are driven by methanotrophy and geological processes influencing fluid composition. Further geochemical and microbiological studies are required to disentangle these intriguing isotopic results, including the measurement of Capelinhos methane concentrations in both end-member and diffuse fluids, and evaluation of the composition of microbial communities compared to those found at other LS edifices. Broadly, our isotopic results: (1) highlight the necessity of characterizing isotopic sources while analyzing multiple isotopes to confidently interpret trophodynamics at chemosynthetic-based habitats, and (2) support the hypothesis that *B. azoricus* and its associated fauna do not depend on specific trophic pathways or food sources, but rather have a great trophic plasticity allowing them to colonize a wide array of contrasting chemical environments, as previously postulated^44,65,75,76^.

## Material and Methods

### Study area, sampling and sample processing

Capelinhos is located ~ 1.5 km to the east of the main Lucky Strike (LS) area (nMAR) at 1665 m depth (37.28917 N, − 32.26388 E)^52^ (Fig. 1A) (not to be confused with the Capelinhos volcano on the Faial Island, Azores Archipelago^77^). The LS hydrothermal vent field (~ 1700 m depth), discovered in 1992, is located on the Lucky Strike Seamount on the Azores Triple Junction^61,78^. It is a basalt-hosted vent field fueled by a magmatic chamber located at 3–3.5 km depth^78,79^. Hydrothermal activity occurs at > 20 sulfide edifices/structures located around a fossil lava lake of ~ 300 m in diameter situated in between three ancient volcanic cones^78,79^. Edifices emit both high-temperature focused-fluids, ranging from 200 to 340 °C, and low-temperature diffuse venting, and show distinct fluid chemical compositions^52,53^. Nevertheless, evidence suggest a common reaction zone for the entire field, including Capelinhos^53^.

Capelinhos is formed by a 20-m high mound discovered in 2009 in the microbathymetric data during the Bathyluck 2009 cruise (10.17600/9030040) ^52^. Hydrothermal activity was only confirmed in 2013, when Capelinhos was visually inspected during the Momarsat 2013 cruise (10.17600/13030040) ^52^. Hydrothermal activity is mainly localized at the mound top, where inactive and active “candelabra”-like chimneys (~ 12 m high) vent focused high-temperature hydrothermal fluids (T = 324 °C) whereas diffuse venting areas are scarce and mainly occur at the mound base^52,53^. Compared to nearby LS vent edifices, Capelinhos shows poorly-altered fluids that are quickly transported from the common LS reaction zone to the seafloor, giving rise to a fluid end-member chemistry with very low pH (2.56) and chloride (262.3 ± 0.1 mM), and high iron (2789.4 ± 84.8 μM) and manganese (639.5 ± 27.6 μM) concentrations (see Table 2 in^53^).

Capelinhos was revisited during the Momarsat 2014 (10.17600/14000300) and 2015 (10.17600/15000200) cruises on board of the R/V *Pourquoi Pas?* which visually inspected chimneys, diffuse flow areas and the periphery using the ROV *Victor6000*. In 2014, three temperature probes were deployed on a large-size mussel bed on a diffuse flow area at the base of one edifice. The probes registered temperatures every 15 min for 9 months, form the 24th July 2014 to the 20th/23rd April 2015. In 2015, prior to faunal sampling associated to mussels, sulfide (ƩS) and iron (Fe(II)) concentrations were measured in situ using the chemical analyzer CHEMINI^80^ over the targeted mussel bed. Biological samples were collected using *Victor6000*’s manipulator arm (three to four grabs per sample) and placed in isotherm boxes. After grabs, a suction sampler was used on each sampling area to collect all remaining fauna. Sampled area was estimated analyzing *Victor6000*’s videos with the software ImageJ. Once on board, samples were sieved through 250 µm (directly fixed in 96° ethanol) and 20 µm (fixed with 4% buffered formalin, then in 96° ethanol, not considered in this study) mesh-sizes. Individuals were sorted, identified to the lowest taxonomic level possible using stereo- and binocular microscopes (except for mussel specimens, which were barcoded to identify the species), and counted (only individuals with complete anterior regions). Meiofaunal organisms (e.g., copepods and nematodes) found in the macrofaunal samples were included in the analyses.

### Genetic analyses

We used the mitochondrial cytochrome oxidase I (mtCOI) to identify mussel specimens. In short, we extracted 10–50 mg of mussel tissue to be digested until total digestion with proteinase K at 60 °C in 0.5 ml pK-CTAB lysis buffer (containing 2% CTAB (Cetiltrimetilamina), 1 M NaCl, 1% PVP (Polyvinylpirrolidina), 20 mM EDTA pH8, 100 mM Tris–HCl pH 8, 0.1 mg mL-1 proteinase K).

Then, we: (1) extracted genomic DNA using the phenol/chloroform protocol, (2) precipitated it with Isopropanol and washes with ethanol 70%, (3) re-suspended and stored the pellet at − 20 °C in molecular quality sterile water until amplifications, (4) obtained partial sequences of the mtCOI gene using the specific primers:

BathCOI-F 5′-GTGGTCTGGAATAATTGGAAC-3′, and

BathCOI-R 5′-ATAAAAAGATGTATTRAARTGACG-3′

following Olu-Le Roy et al.^81^, and (5) amplified the DNA as follows: an initial step of denaturation at 94 °C for two minutes, five cycles of 35 s/94 °C, 35 s/48 °C and 70 s/72 °C, thirty-five cycles of 35 s/94 °C, 35 s/52 °C, 70 s/72 °C, and an final elongation at 72 °C for 10 min. We perform PCR reactions into a 25-ml reaction volume (1X PCR buffer, 2,2 mM MgCl_2_, 0.5 mM of each dNTPs, 0.55 µM of each primer, 0.02 U of *Taq* polymerase (GoTaq Promega), and 20 ng genomic DNA). The PCR products were purified and sequenced using the 3730XL Sequencer by thermofisher (Macrogen Europe, The Netherlands) following the manufacturer’s protocol.

### Biodiversity and community composition

We estimated species diversity by individual-based rarefaction of Hill numbers (D)^82^ of *q* orders 0, 1 and 2, respectively. D expresses diversity in effective species number, i.e., the number of equally-common species that would represent the observed diversity, thus overcoming the problems of expressing indexes in different scales and respecting the “replication principle”^82–84^. When *q* = 0, ^0^D is the species number giving equal weight to abundant and rare species^82,83^. When q = 1, ^1^D is the exponential of Shannon index^85^ weighting species proportionally to their abundances and providing the effective number of common species in the assemblage^82,83^. When q = 2, ^2^D is the Simpson index^86^, which gives more weight to the dominant species and provides the effective number of dominant species in the assemblage^82,83^. We compared Capelinhos rarefied diversities (^0^D, ^1^D and ^2^D) to those from Cypress, Y3, Eiffel Tower and Montségur (LS), Atos (Menez Gwen), Thermitière and France (Rainbow) edifices using raw data from Sarrazin et al.^44^. Prior to comparisons, we removed copepod, nematode and ostracod taxa since we identified them at higher taxonomic resolutions than in previous studies. We estimated rarefaction curves and 95% confidence intervals for 1050 individuals and then plotted them using the *iNEXT* function of the *iNEXT* package^87^ in R V.4.0.2 environment^88^.

We compared Capelinhos species composition to those of LS, Menez Gwen, Broken Spur, Rainbow, TAG, Snake Pit, Logatchev and Ashadze-1 nMAR vent fields to disentangle biogeographic affinities based on the presence/absence dataset (including meiofauna) provided by Boschen-Rose and Colaço^41^. For consistency, we removed all taxa not identified at the species level. Moreover, we updated this dataset by including the annelid *Ophryotrocha fabriae* Paxton & Morineaux, 2009 and the nematode *Oncholaimus dyvae* Zeppilli et al., 2019, since both species inhabit the LS vent field^55^. We then computed a β-diversity dissimilarity distance matrix using Simpson’s pairwise dissimilarity metric (β_sim_), which ranges between 0 (no species compositional differences) to 1 (totally dissimilar species composition)^89^. β_sim_ is independent of species richness differences and thus, only accounts for species turnover: it does not identify natural species-poor or unevenly-sampled communities as being highly dissimilar^89,90^. We computed β_sim_ using the function *beta.pair* in the package *betapart* in R^91^. We performed a hierarchical cluster analysis (HCA) using the Average-linkage cluster algorithm, the β_sim_ dissimilarity distance matrix and a multiscale bootstrap resampling to calculate the Approximately Unbiased p-values (AU-*P*) to identify robust clusters, with significant AU-*P* being set at > 95^92^. We estimated the bootstrap by repeatedly and randomly sampling sites performing the HCA by using the function *pvclust* in the package *pvclust* in R^92^.

### Stable isotope analyses

We measured the carbon, nitrogen and sulfur isotopic ratios of 58 samples from 20 species/morphospecies (Appendix: Table S1), either using muscle tissue fragments (for mussels and shrimps), whole specimens or pools of specimens (Appendix: Table S1). For large species/individuals, we manually removed shells to avoid inorganic carbon bias. For smaller organisms with shells (e.g., the gastropods *Protolira valvatoides* Warén & Bouchet, 1993 and *L. atlanticus* Warén & Bouchet, 2001), we placed individuals in tin cups and acidified by direct addition of hydrochloric acid (HCl 1 M) 50 µl increments until detecting no bubbling^93^. We analyzed the isotopes at the University of Liege (Belgium) using a vario MICRO cube (Elementar, Germany) elemental analyzer coupled to an IsoPrime100 (Elementar, United Kingdom) isotope ratio mass spectrometer. We expressed isotope ratios using the δ notation^94^, in ‰ and relative to the international references: Vienna Pee Dee Belemnite (carbon), Atmospheric Air (nitrogen) and Vienna Canyon Diablo Troilite (sulfur). We used as primary analytical standards the following International Atomic Energy Agency (IAEA, Vienna, Austria) certified reference materials: sucrose (IAEA-C-6; δ^13^C = − 10.8 ± 0.5‰; mean ± SD), ammonium sulphate (IAEA-N-1; δ^15^N = 0.4 ± 0.2‰; mean ± SD), and silver sulfide (IAEA-S-1 δ^34^S = − 0.3‰). We used Sulfanilic acid (Sigma-Aldrich; δ^13^C = − 25.6 ± 0.4‰; δ^15^N = − 0.13 ± 0.4‰; δ^34^S = 5.9 ± 0.5‰; means ± SD) as a secondary analytical standard. Standard deviations on multi-batch replicate measurements of secondary and internal lab standards (seabass muscle) interspersed with samples (one replicate of each standard every 15 analyses) were 0.2‰ for both δ^13^C and δ^15^N and 0.4‰ for δ^34^S.

We used isotopic ratios to discuss the potential energy acquisition pathways of fauna and to compare the trophic structure with the close, well-characterized Eiffel Tower edifice at the main LS. At LS, δ^13^C values reflect methane oxidation, i.e., methanotrophy (− 12.9 ± 3.4‰) and/or sulfide oxidation, i.e., thiotrophy (− 36 to − 30‰, when using Calvin-Benson-Bassham cycle; − 15 to − 10‰, when using the reductive tricarboxylic acid cycle)^65^. Nitrogen reflects the use of nitrates (δ^15^N = 5–7‰) and/or ammonium (δ^15^N < 0‰)^95,96^. Particularly at LS, photosynthesis-derived organic matter range from − 24 to − 22‰ δ^13^C and from 4 to 6‰ δ^15^N^69^. δ^34^S values around or below 10‰ discern organic matter of chemosynthetic origin and, around or over 16‰, of photosynthetic origin^72^. We obtained the isotope values of Eiffel Tower assemblages from Alfaro-Lucas et al.^56^.

### Supplementary Information

Supplementary Information 1.

Supplementary Video 1.

## Data Availability

Data are publicly available from the SEANOE repository (DOI: 10.17882/90421).
