# Supplementary material for: Fluid chemistry alters faunal trophodynamics but not composition on the deep-sea Capelinhos hydrothermal edifice (Lucky Strike vent field, Mid-Atlantic Ridge)
Source: Sci Rep. 2024 Jan 22;14:1940. doi: 10.1038/s41598-024-52186-1 (PMC10803789; doi:10.1038/s41598-024-52186-1)
Supplement: Supplementary file 1 — Supplementary Information 1. [file 41598_2024_52186_MOESM1_ESM.docx]

**Supplementary information**

**Fluid chemistry alters faunal trophodynamics but not composition on the deep-sea Capelinhos hydrothermal edifice (Lucky Strike vent field, Mid-Atlantic Ridge)**

Joan M. Alfaro-Lucas^1, 4, *^, Daniel Martin^2^, Loïc N. Michel^1, 3^, Agathe Laes^1^, Cécile Cathalot^1^, Sandra Fuchs^1^, Jozée Sarrazin^1^

^1^ Univ Brest, Ifremer, CNRS, Unité BEEP, F-29280 Plouzané, France.

^2^ Centre d’Estudis Avançats de Blanes (CEAB-CSIC), Blanes, Catalonia, Spain.

^3^ Université de Liège, Liège, Belgium.

^4^ Current address: Department of Biology, University of Victoria, Victoria, British Columbia, Canada.

| **Table S1**. δ¹³C, δ¹^5^N and δ^34^S stable isotope compositions (‰) of species found on the Capelinhos edifice (off main Lucky Strike vent field). Tissue = analyzed tissue. n = number of individuals. Acidification = acidification for carbonate removal. N.D.: under range. | | | | | | | |
| --- | --- | --- | --- | --- | --- | --- | --- |
| **Species** | **Tissue** | **n** | **Dive** | **Acidification** | **δ¹³C** | **δ¹⁵N** | **δ³⁴S** |
| *Alvinocaris markensis* | Abdominal muscle | 1 | 604 | - | -23.47 | 2.79 | 8.55 |
| Amphipoda sp. 1 | Whole body | 1 | 604 | - | -18.75 | 3.27 | N.D. |
| *Amphisamytha lutzi* | Whole body | 1 | 602 | - | -19.17 | 1.66 | 12.08 |
| *Amphisamytha lutzi* | Whole body | 1 | 602 | - | -17.60 | 2.30 | 11.52 |
| *Amphisamytha lutzi* | Whole body | 1 | 602 | - | -17.55 | 1.96 | 11.91 |
| *Amphisamytha lutzi* | Whole body | 1 | 602 | - | -17.24 | 2.88 | 10.59 |
| *Amphisamytha lutzi* | Whole body | 1 | 602 | - | -17.74 | 3.19 | 11.68 |
| *Amphisamytha lutzi* | Whole body | 1 | 604 | - | -14.74 | 4.30 | 10.77 |
| *Amphisamytha lutzi* | Whole body | 1 | 604 | - | -16.91 | 4.76 | 10.32 |
| *Amphisamytha lutzi* | Whole body | 1 | 604 | - | -15.58 | 4.59 | 11.67 |
| *Amphisamytha lutzi* | Whole body | 1 | 604 | - | -13.07 | 6.40 | 10.47 |
| *Amphisamytha lutzi* | Whole body | 1 | 604 | - | -16.97 | 3.15 | 11.33 |
| *Amphisamytha lutzi* | Whole body | 1 | 604 | - | -17.89 | 2.22 | 8.52 |
| *Amphisamytha lutzi* | Whole body | 1 | 604 | - | -18.43 | 0.30 | 11.66 |
| *Amphisamytha lutzi* | Whole body | 1 | 604 | - | -17.94 | 4.11 | 11.61 |
| *Amphisamytha lutzi* | Whole body | 1 | 604 | - | -18.82 | 1.18 | 13.25 |
| *Amphisamytha lutzi* | Whole body | 1 | 604 | - | -17.69 | 3.04 | 11.45 |
| Dirivultidae *sp.* | Whole body | ~180 | Pool | - | -19.48 | 0.49 | 9.67 |
| Dirivultidae *sp.* | Whole body | ~180 | Pool | - | -19.25 | 1.00 | 9.13 |
| *Bathymodiolus azoricus* | Foot muscle | 1 | 604 | - | -23.68 | -7.26 | 6.43 |
| *Bathymodiolus azoricus* | Foot muscle | 1 | 604 | - | -24.75 | -7.31 | 6.04 |
| *Bathymodiolus azoricus* | Foot muscle | 1 | 604 | - | -18.59 | -4.84 | 7.26 |
| *Bathymodiolus azoricus* | Foot muscle | 1 | 602 | - | -17.07 | -0.72 | 10.16 |
| *Bathymodiolus azoricus* | Foot muscle | 1 | 602 | - | -19.26 | -3.07 | 9.46 |
| *Bathymodiolus azoricus* | Foot muscle | 1 | 602 | - | -22.36 | -5.01 | 8.44 |
| *Bouvierella curtirama* | Whole body | 3 | 604 | - | -19.05 | 3.16 | 9.28 |
| *Bouvierella curtirama* | Whole body | 3 | 604 | - | -19.06 | 2.44 | 12.53 |
| *Branchinotogluma sp.* | Whole body | 1 | 602 | - | -24.91 | 6.93 | 10.11 |
| *Branchinotogluma sp.* | Whole body | 1 | 602 | - | -16.17 | 7.59 | 11.27 |
| *Branchinotogluma sp.* | Whole body | 1 | 602 | - | -29.76 | 5.52 | 9.32 |
| *Branchipolynoe seepensis* | Whole body | 1 | 604 | - | -18.65 | -0.55 | 8.24 |
| *Branchipolynoe seepensis* | Whole body | 1 | 604 | - | -15.85 | 2.92 | 8.94 |
| *Branchipolynoe seepensis* | Whole body | 1 | 604 | - | -23.72 | -7.05 | 8.14 |
| *Branchipolynoe seepensis* | Whole body | 1 | 602 | - | -23.62 | -5.68 | 8.76 |
| *Branchipolynoe seepensis* | Whole body | 1 | 602 | - | -21.34 | -3.14 | 9.15 |
| *Branchipolynoe seepensis* | Whole body | 1 | 602 | - | -21.34 | -5.07 | 9.03 |
| *Branchipolynoe sp.* | Whole body | 1 | 602 | - | -19.18 | -1.15 | 9.72 |
| *Divia briandi* | Whole body w/o shell | 10 | Pool | - | -11.89 | 5.95 | 8.29 |
| *Glycera tesselata* | Whole body | 1 | 604 | - | -21.84 | 9.11 | N.D. |
| *Lepetodrilus atlanticus* | Whole body | 21 | Pool | yes | -20.04 | 4.30 | 12.17 |
| *Luckia striki* | Whole body | 2 | 604 | - | -20.18 | 4.46 | N.D. |
| *Mirocaris fortunata* | Abdominal muscle | 1 | 604 | - | -18.67 | 5.46 | 9.92 |
| *Mirocaris fortunata* | Abdominal muscle | 1 | 604 | - | -17.41 | 5.60 | 9.39 |
| *Mirocaris fortunata* | Abdominal muscle | 1 | 602 | - | -17.84 | 5.31 | 10.47 |
| *Mirocaris fortunata* | Abdominal muscle | 1 | 604 | - | -14.32 | 6.84 | 10.14 |
| *Mirocaris fortunata* | Abdominal muscle | 1 | 604 | - | -15.58 | 6.67 | 10.13 |
| *Mirocaris fortunata* | Abdominal muscle | 1 | 604 | - | -14.27 | 6.87 | 10.14 |
| *Mirocaris fortunata* | Abdominal muscle | 1 | 604 | - | -19.33 | 7.60 | 15.04 |
| *Mirocaris fortunata* | Abdominal muscle | 1 | 604 | - | -17.44 | 4.78 | 9.87 |
| *Mirocaris fortunata* | Abdominal muscle | 1 | 604 | - | -16.87 | 6.95 | 8.98 |
| *Mirocaris fortunata* | Abdominal muscle | 1 | 604 | - | -15.47 | 7.33 | 9.30 |
| Nemertea sp. | Whole body | 8 | Pool | - | -17.23 | 2.04 | 10.85 |
| *Oncholaimus dyvae* | Whole body | 171 | Pool | - | -18.32 | 6.32 | 9.51 |
| *Ophryotrocha fabriae* | Whole body | 53 | Pool | - | -24.88 | 2.55 | N.D. |
| *Protolira valvatoides* | Whole body | 13 | Pool | yes | -22.98 | 3.97 | 11.97 |
| *Pseudorimula midatlantica* | Whole body w/o shell | 12 | Pool | - | -25.63 | 2.25 | 8.11 |
| *Pseudorimula midatlantica* | Whole body w/o shell | 13 | Pool | - | -26.35 | 2.30 | 7.99 |
| *Sericosura* sp. | Whole body | 1 | 604 | - | -21.57 | 4.61 | 10.35 |

| **Table S2.** Concentrations of iron (Fe(II)) and sulfide (ƩS), and mean associated temperatures during measurements (Fe(II)-T**/** ƩS -T), on the three *Bathymodiolus azoricus* mussel bed samples of the Capelinhos sulfide structure located ~1.5 km off the main Lucky Strike vent field (northern Mid-Atlantic Ridge). Iron and sulfide concentrations associated to samples PL604 C2 and PL602 C3 were averaged from 2 and 3 measurements, respectively. Iron and sulfide concentrations associated to sample PL604 C1 and one PL604 C2 were contaminated and not considered. | | | | |
| --- | --- | --- | --- | --- |
| **Sample** | **Fe(II)** | **Fe(II)-T (ºC)** | ƩS | ƩS **-T (ºC)** |
| PL604 C1 | - | - | - | - |
| PL604 C2 | - | - | - | - |
| PL604 C2 | 1.66 | 4.40 ± 0.24 | 44.28 | 4.51 ± 0.29 |
| PL602 C3 | 0.98 | 4.63 ± 0.93 | 33.06 | 4.60 ± 1.01 |
| PL602 C3 | 1.74 | 4.40 ± 0.87 | 28.36 | 4.46 ± 0.96 |
| PL602 C3 | 2.40 | 5.59 ± 1.19 | 21.80 | 6.58 ± 0.89 |

| 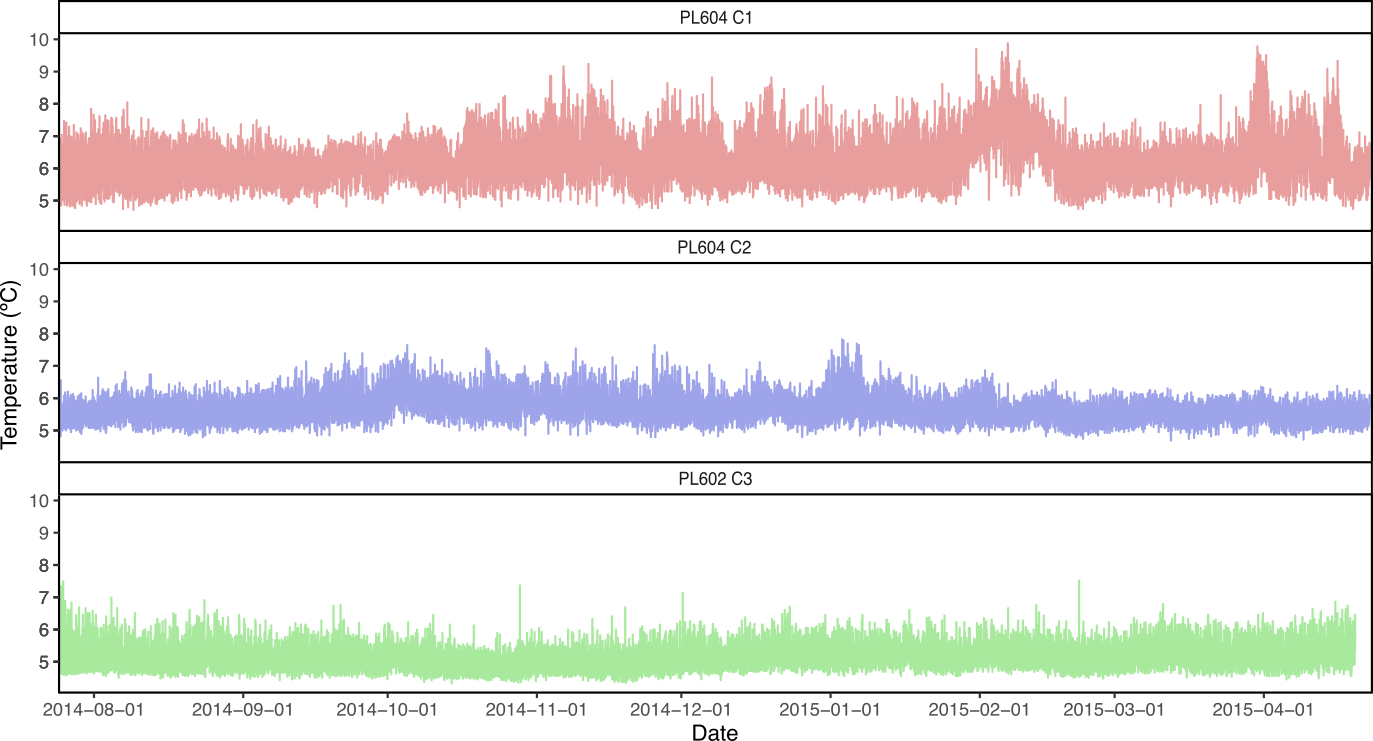 |
| --- |

**Figure S1**. Temperature recorded associated with the three samples of *Bathymodiolus azoricus* mussel assemblages of Capelinhos. Temperature was recorded every 15 minutes during 9 months using autonomous probes deployed on the three sampled assemblages.


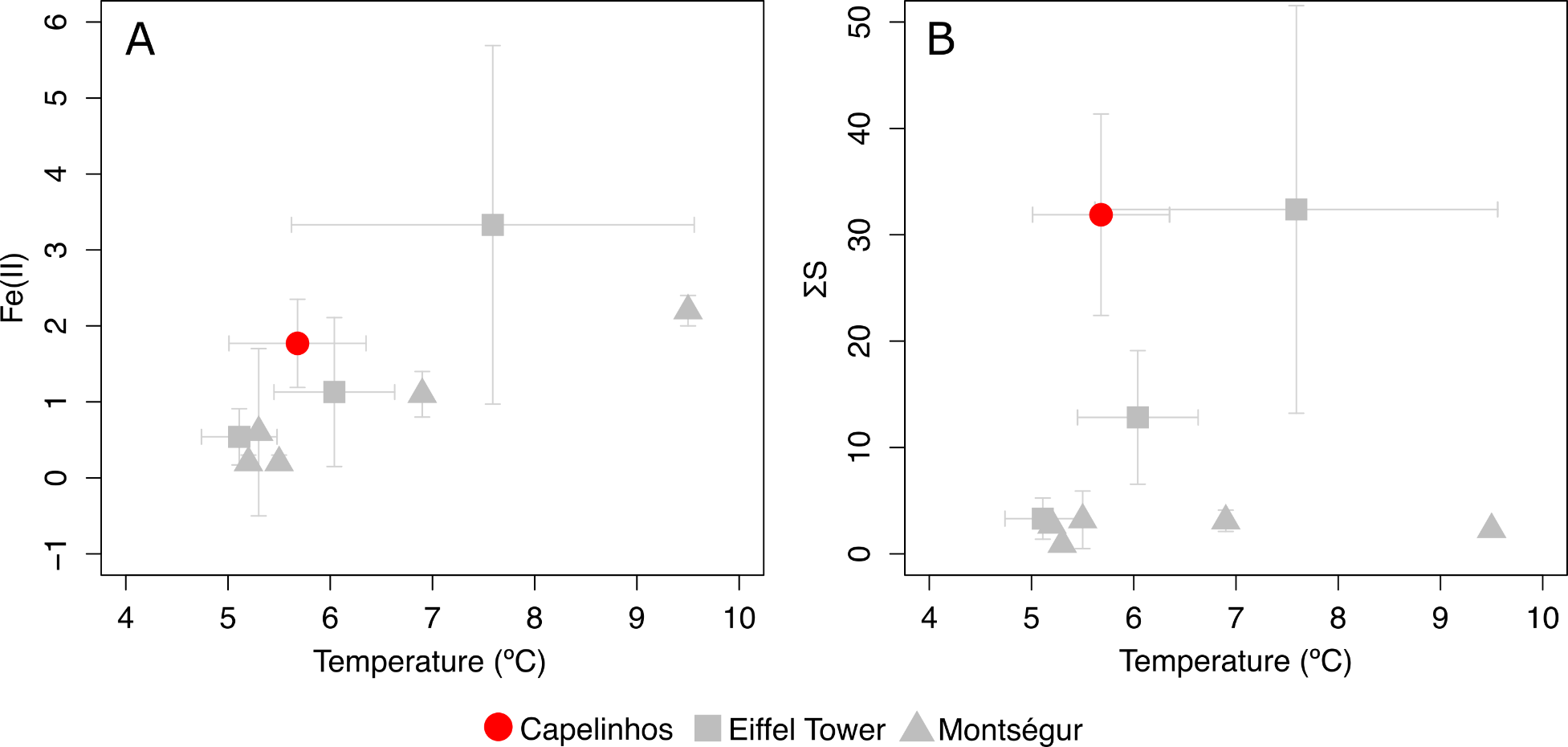


**Figure S2**. Concentrations of iron (Fe(II)) (A) and sulfide (ƩS) (B) in relation to temperatures (mean ± SD) at Capelinhos, Eiffel Tower and Montségur edifices (Lucky Strike vent field, northern Mid-Atlantic Ridge) on *Bathymodiolus azoricus* mussel bed samples. Data from this study (Capelinhos), Sarrazin et al. (2015) (Eiffel Tower), and Marticorena et al. (2021) (Montségur).

| **Table S3**. Raw abundance (number of individuals) of species in the three mussel assemblage samples obtained at Capelinhos edifice (off main Lucky Strike vent field). Sampled areas for sample PL604 C1, PL604 C2 and PL602 C3 were 0.23, 0.23 and 0.33 m^2^, respectively. | | | | | | |
| --- | --- | --- | --- | --- | --- | --- |
| **Phylum** | **Class** | **Order** | **Species** | **PL604 C1** | **PL604 C2** | **PL602 C3** |
| Annelida | Polychaeta | Terebellida | *Amphisamytha lutzi* | 233 | 66 | 81 |
|  |  | Phyllodocida | *Glycera tesselata* | 1 | 0 | 0 |
|  |  |  | *Branchipolynoe seepensis* | 101 | 14 | 69 |
|  |  |  | Polynoidae sp. 2 | 4 | 0 | 0 |
|  |  |  | Polynoidae sp. 3 | 5 | 0 | 5 |
|  |  |  | *Branchinotogluma* sp. | 6 | 1 | 3 |
|  |  |  | *Branchinotogluma* sp. 2 | 1 | 0 | 0 |
|  |  |  | Hesionidae sp. | 0 | 2 | 0 |
|  |  | Eunicia | *Ophryotrocha fabriae* | 32 | 5 | 17 |
| Arthropoda | Hexanauplia | Harpacticoida | *Smacigastes micheli* | 7 | 1 | 0 |
|  |  |  | Ameiridae sp. 1 | 8 | 36 | 29 |
|  |  |  | Miraciidae sp. | 4 | 28 | 8 |
|  |  | Siphonostomatoida | Dirivultidae sp. | 226 | 48 | 103 |
|  | Malacostraca | Amphipoda | *Luckia striki* | 3 | 0 | 0 |
|  |  |  | *Bouvierella curtirama* | 12 | 3 | 0 |
|  |  |  | Amphipod sp. 1 | 1 | 0 | 0 |
|  |  | Decapoda | *Mirocaris fortunata* | 21 | 21 | 2 |
|  |  |  | *Alvinocaris markensis* | 0 | 1 | 0 |
|  | Ostracoda |  | Ostracoda sp. | 13 | 11 | 18 |
|  | Pycnogonida | Pantopoda | *Sericosura sp.* | 0 | 2 | 0 |
| Mollusca | Bivalvia | Mytilida | *Bathymodiolus azoricus* | 289 | 62 | 110 |
|  | Gastropoda | Lepetellida | *Lepetodrilus atlanticus* | 10 | 10 | 3 |
|  |  |  | *Pseudorimula midatlantica* | 28 | 2 | 12 |
|  |  | Trochida | *Protolira valvatoides* | 7 | 5 | 1 |
|  |  |  | *Lurifax vitreus* | 1 | 0 | 0 |
|  |  | Cycloneritida | *Divia briandi* | 10 | 0 | 0 |
| Nematoda | Enoplea | Enoplida | *Oncholaimus dyvae* | 128 | 8 | 40 |
| Nemertea |  |  | Nemertea sp. | 2 | 5 | 1 |

**References**

Sarrazin, J. *et al.* Biodiversity patterns, environmental drivers and indicator species on a high-temperature hydrothermal edifice, Mid-Atlantic Ridge. *Deep Sea Research Part II: Topical Studies in Oceanography* 121, 177–192 (2015).

Marticorena, J. *et al.* Recovery of hydrothermal vent communities in response to an induced disturbance at the Lucky Strike vent field (Mid-Atlantic Ridge). *Mar Environ Res* 168, 105316 (2021).
